# Federating patients identities: the case of rare diseases

**DOI:** 10.1186/s13023-018-0948-6

**Published:** 2018-11-12

**Authors:** Meriem Maaroufi, Paul Landais, Claude Messiaen, Marie-Christine Jaulent, Rémy Choquet

**Affiliations:** 1 Banque Nationale de Données Maladies Rares, Hôpital Necker Enfants Malades, Assistance Publique des Hôpitaux de Paris, Paris, France; 2 INSERM, U1142, and UMR_S 1142, LIMICS, Sorbonne University, Paris, France; 3 Pierre and Marie Curie University, Paris, France; 4 Paris 13 University, F-93430 Villetaneuse, France; 5 UPRES EA2415, Clinical Research University Institute, Montpellier University, 641 avenue du Doyen Gaston Giraud, 34093 Montpellier, France; 6 INSERM UMRS 933, Rare Disease Cohorts (RaDiCo), Sorbonne University, and Hôpital Trousseau, Assistance Publique Hôpitaux de Paris, Paris, France

**Keywords:** Rare diseases, Health information exchange, Patient identification systems, Identity federation, Patient data privacy

## Abstract

**Background:**

Patient information in rare disease registries is generally collected from numerous data sources, necessitating the data to be federated. In addition, data for research purposes must be de-identified. Transforming nominative data into de-identified data is thus a key issue, while minimizing the number of identity duplicates. We propose a method enabling patient identity federation and rare disease data de-identification while preserving the pertinence of the provided data.

**Results:**

We developed a rare disease patient identifier. The IdMR generation process is a three-phased algorithm involving a hash function to irreversibly de-identify nominative patient data, including those of foetuses. This process minimizes collision risks and reduces variability for the purpose of identity federation. The IdMR was generated for 360,000 patients of the CEMARA database. It allowed identity federation of 1771 duplicated files. No collisions were introduced.

**Conclusion:**

We examined and discussed the risks of collisions and the creation of duplicates as well as the risks of patient re-identification. We discussed our choice of nominative input information in light of that used by other patient identification solutions. The IdMR is a patient identifier that enables identity federation and file linkage. The simplicity of the algorithm and the universality and stability of the input data make it a good candidate for European cross-border rare disease projects.

## Introduction

A rare disease (RD) affects, by definition, a small number of patients, with a prevalence of no more than 5/10,000 in Europe [1] and 7.5/10,000 in the USA [2]. Between 7000 and 8000 [3] existing RDs may affect a total of 3 to 4 million people in France, representing 4.5 to 6% of the total French population [4]. About 30 million people are affected in Europe [5] and 25 million in the USA [6].

The French ministry of health launched the French Rare Diseases Registry project, called the BNDMR project (Banque Nationale de Données Maladies Rares), in 2011. It aims to build a national RD registry system. It is designed to record clinician activities and facilitate socioeconomic and epidemiological RD studies through the identification of RD patients. This national information system collects RD patient data on the basis of a minimum dataset [7] and promotes data exchange with existing systems to avoid data re-entry by health professionals. Interoperability issues are a major ongoing challenge. Data standardization and mapping detection is necessary to make the data comprehensible by the transmitter and receiver systems [8]. Moreover, an identity federation mechanism capable of managing de-identified data from several sources of information that avoids duplicates or identity collisions is essential to collect appropriate data on rare cases.

**Fig. 1:**
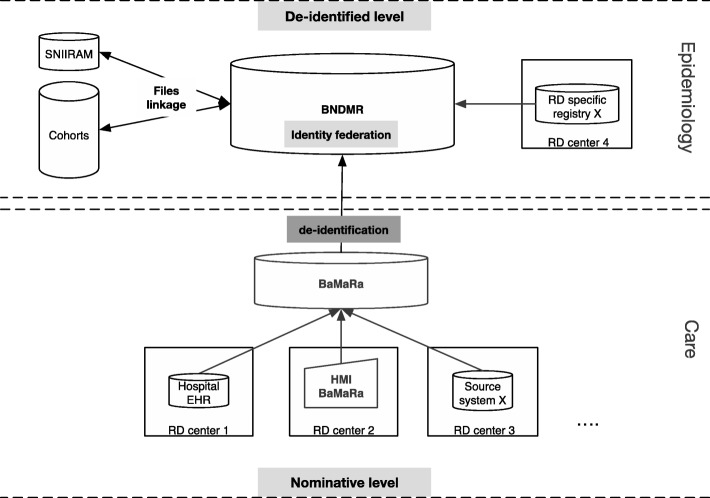
The French rare diseases information system architecture

The BNDMR information system has a two-layered architecture (Fig. 1) that generates patients’ identifiers constraints. The first is a data entry web application, BaMaRa that uses nominative data to ensure patient follow-up at the point of care. The collected data is periodically de-identified, consolidated, and transferred to the second layer, which is a de-identified data warehouse that allows statistical and epidemiological studies. Interoperability had to be ensured at both levels to allow interconnections between different types of source systems.

The first layer of the BNDMR is interconnected to the hospital electronic health records through nominative data related to patients’ follow-up in a medical care perspective. Interconnections at the nominative level are not allowed for other source systems, e.g. RD specific registries. Indeed, the legally declared objectives of the systems to be connected are not the same: e.g. medical care follow-up versus epidemiological objective. In this latter case, these systems are thus connected at a de-identified level with the data warehouse. A reliable patient identifier must be used for both levels to avoid creating duplicates in the database, especially at the de-identified level, where the patient identifier has to be assigned to the data by the source system.

The patients’ identifier must be non-meaningful. There must be no personal information since the national registry is a de-identified database. A unique and permanent identifier has to be attributed to every patient regardless of his/her medical care pathway in the healthcare institutions. This identifier needs to be unique for a given patient in order to avoid collisions. Children and even foetuses, which represent a large proportion of RD patients (14) [9], must have their own identifiers. Indeed, 80% of RDs are genetic and usually appear during childhood [4].

French authorities chose the national social security number (NIR) as the new national health identifier [10]. It is generated at birth (or at entry in the country for non-French residents) and is managed by the national identification directory for the identification of natural persons (RNIPP). This identification number is unique to the individual. It is displayed on the national health insurance card and used by social security beneficiaries for identification and reimbursement of health spending. However, children do not have a personal social security card. They are registered on their parent’s card but their personal NIR is not listed. The parent’s NIR is used to reimburse the healthcare expenses of their children. This is an important limitation of using the NIR to identify RD patients since most are diagnosed in childhood.

Our objective was to propose a new national RD patient identifier, the IdMR (Identifiant Maladies Rares), built for the French RD registry, but that remains universal in its design. It is illustrated with data from the French RD Registry. This patient identifier must prevent the risk of patient re-identification and allow the federation of patient identities, especially for epidemiological and statistical purposes.

## Methods

The new identifier for the French RD registry relies on nominative data (i.e. person identity), which are, to date, the most widespread and reliably available information.

Four types of input data were used to create the patient identifier: 1) the first name among the names listed on the birth certificate; 2) the family name, also called patient’s birth name or patronymic name as reported on the birth certificate; 3) the complete date of birth: day, month, and year; 4) the legal sex: male, female, or indeterminate.

Foetuses that are not yet legally registered may have neither a first name nor a family name, they do not yet have a birth date, and their gender may not yet be known. One solution may be to create an extra file to link the file of the foetus to the mother’s identity. However, this would introduce a new functionality in the application and would be complicated for some source systems. A simpler solution was to consider foetuses as patients like the others, but to create their nominative data differently.

### A: Data pre-processing

The first step in IdMR generation is the pre-processing of these four pieces of nominative data. The data is collected from different sources, resulting in potential variability introduced by typing errors or minor variations of spelling.

#### Format formalization

The date of birth respects the ISO-8601 format (without dashes), 8 numeric characters in the YYYYMMDD format. Alphabetic characters are used for gender: the character “F” for female patients, “M” for male patients, and “I” for patients of indeterminate gender. It is important to provide users with this possibility, especially for foetuses and in case of diseases affecting the genital system, even if the indeterminate gender is not officially recognized.

#### Free text processing

Only alphanumeric characters are allowed (UTF-8 encoding ASCII compatibility) for the first and last name: alphabetic characters A-Z and numeric characters representing the numbers 0 to 9. Accented characters are replaced by the corresponding non-accented characters. Any special characters (symbols, spaces and punctuation) are deleted. All lowercase alphabetic characters are replaced by the corresponding uppercase characters.

**Fig. 2:**
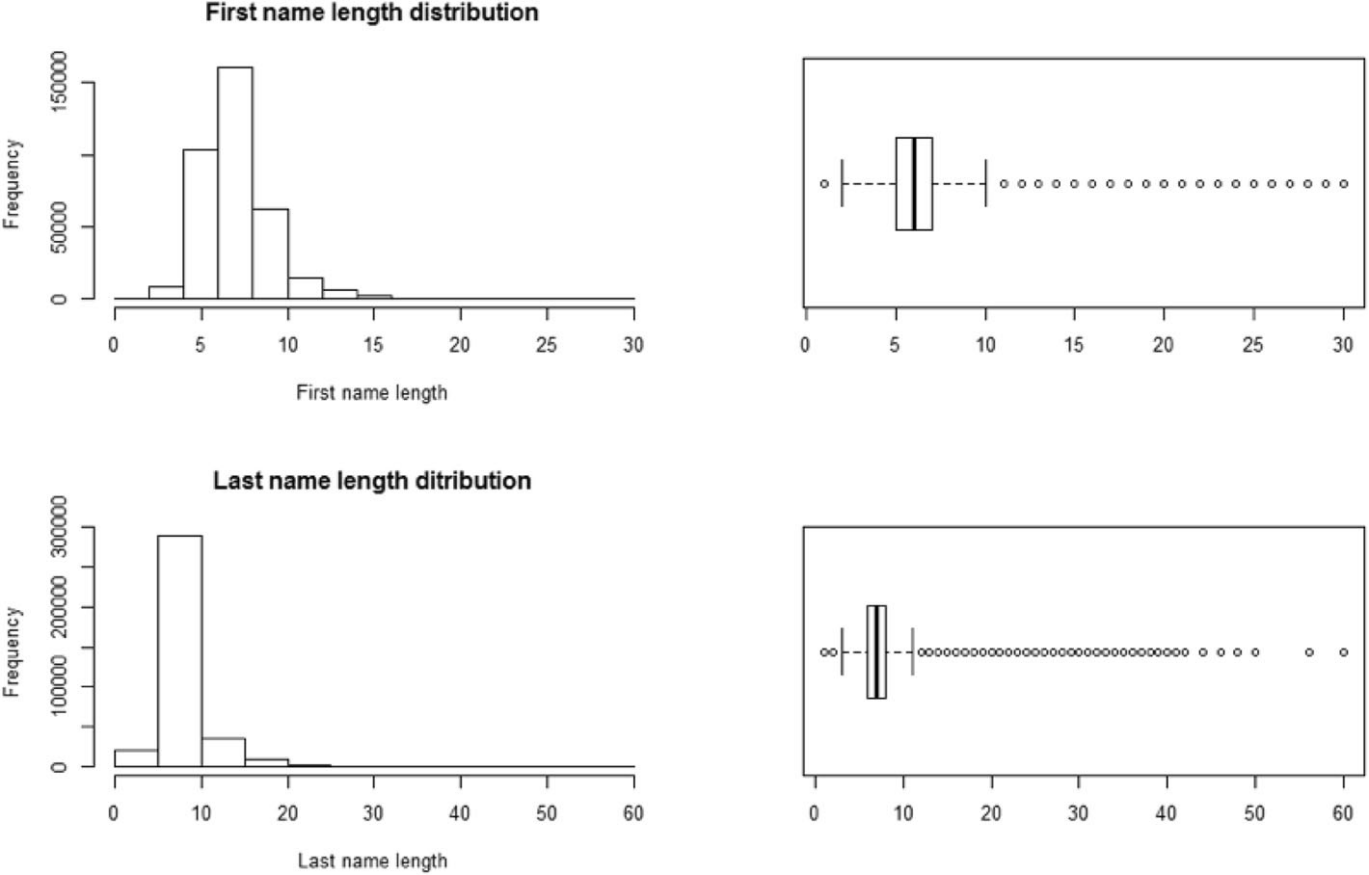
First names and last names lengths distributions

We conducted a study of the distribution of the length of first and last names for 280,000 identities to determine the threshold to be applied to the length of the data (Fig. 2). The median was 6.5 characters for the first names and 7.1 characters for the last names. We chose a 10-character threshold of each to give 75% coverage of the studied population. Thus, the first and last names are truncated not to exceed 10 characters each. If the threshold is not reached, spaces are added on the right to fill up all 10 spaces.

For a foetus, the letter “f” (standing for foetus) is stored as the first name, followed by the rank of sibs in the case of twins, concatenated to the mother’s first name; e.g. f1Marta for Marta’s foetus 1, f2Marta for foetus 2. The mother’s maiden name is stored as the foetus family name. The date of birth is replaced by the date of early pregnancy by using the year and month, and the day is set to the first of the month; e.g. if the date of early pregnancy is estimated to be November 11, 2014, the date used for the IdMR calculation becomes November 1, 2014. This reduces the risk of error due to an approximate date of pregnancy while enabling its identification. The gender of the foetus is set to “I” (standing for indeterminate) for all foetuses, even if the gender is already known. Thus, the IdMR remains the same for the foetus during the entire pregnancy and the gender does not evolve from indeterminate to male or female.

This identification method for foetuses enables generation of the IdMR for foetuses and federation of their files when they are seen in different centres of expertise.

### B: Hashing

Fields containing the processed data are concatenated without adding separators respecting the following order: first name, last name, date of birth, and gender. We obtain a primary string of 29 characters containing nominative information.

This primary string must be transformed into a non-significant string that remains unique for each patient to reduce patient re-identification risk. Hash functions allow this transformation and grant irreversibility unless a mapping table is used to link the input data to the generated hash codes. The use of the Secure Hash Algorithm SHA-256 [11], defined in the FIPS Publication 180–4 by the National Institute of Standards and Technology (NIST) in the United States [12] is recommended in France and is part of the General Security Toolkit published by the National Agency for the Security of Information Systems (ANSSI) [13]. The hash function SHA-256 is used to transform the primary string of 29 characters into a 256-bit hash code.

### C: Hash code post-processing

To enhance its usability, the hash code of 256 bits is converted to decimals. Each byte (8 bits) of the 32 bytes generated by SHA-256 is “translated” into a decimal number (a value from 0 to 255). All leading zeros in these numbers are deleted (25 instead of 025). The 32 obtained numbers are concatenated into a string. The string is truncated to the first 20 characters (left), resulting in the patient identifier.

### D: Collisions

A collision^1^ has been introduced when the number of duplicate identifiers in the output data, the set of generated IdMRs in our case, is greater than the number of duplicate identities in the input data. It is equivalent to there being fewer distinct patients in the output data than in the input data. Collision detection is made at two levels of the IdMR algorithm to detect collisions introduced by the hash function and post-processing (B and C phases) and collisions due to the original nominative data pre-processing (phase A).

**Fig. 3:**
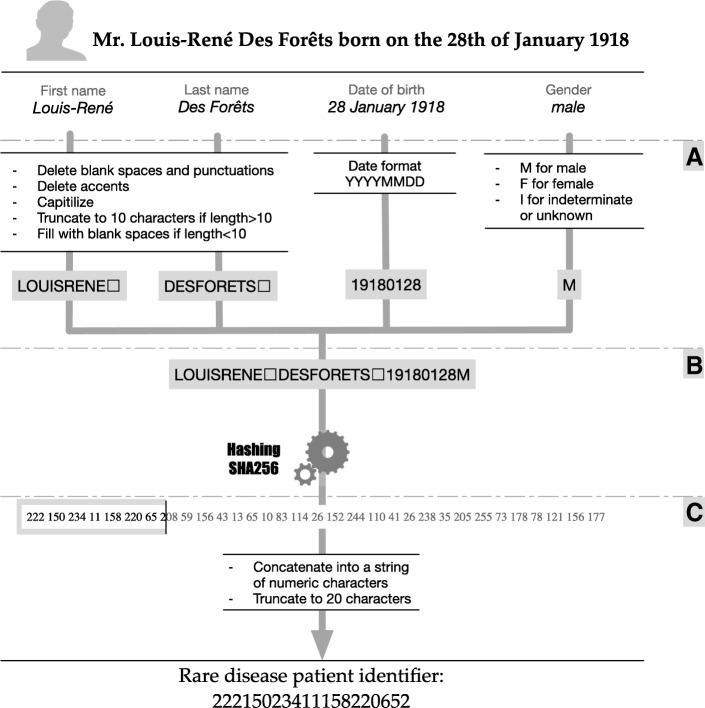
The IdMR algorithm : a three phase process. **a** - Data processing; **b** - Hashing; **c** - Hash code post-processing

In summary, the IdMR generation is a three-phased process (Fig. 3): 1) the nominative data is preprocessed to reduce variability and avoid creating duplicates (same patient with different identifiers); 2) a hash function is used to ensure identifier anonymity; and 3) the resulting hash code is processed to enhance its usability.

## Results

The IdMR was generated for all patient files of CEMARA, a RD database containing 359,339 files (patients and family members) with an estimated duplication rate of approximately 9%. Patients’ data in the database were collected from the first trimester of 2007 to the first trimester of 2015. The input data of the IdMR algorithm (first name, last name, birth date, and gender) are required items in CEMARA.

As stated above, the collision detection was performed at two levels of the IdMR algorithm. First, we estimated the number of collisions introduced by the phase A of the algorithm which is the input nominative data preprocessing. There were fewer exact duplicates in the original nominative data than in the phase A output duplicates. In total, 1771 collisions were detected. We investigated these duplicates by picking a random set of files in which collisions had been detected. We determined that they were patient duplicates with more than one file in the database. This was mostly due to the use of different accents, extra spaces, the use of special characters for compound first names, and the use of either simple or compound versions of family names. The input data preprocessing, and therefore the IdMR algorithm, functioned as an identity federator for these 1771 cases, as it allowed file deduplication.

Second, the same number of duplicates for the strings resulting from the pre-processing phase and their corresponding IdMR were detected. Thus, no collisions were introduced by phases B and C.

Amongst the 359,339 distinct patient files in the CEMARA database, 17,470 exact duplicates were detected using the four nominative input elements of the IdMR algorithm as entered by the users, as well as 1771 supplementary duplicates following the pre-processing phase. The IdMR algorithm detected and allowed identity federation of a total of 19,241 patients, representing 5.35% of the total number of patients’ files in CEMARA (Table 1).

**Table 1:** IdMR generation results for the CEMARA database patients

	Number of duplicates	Percentage	Conclusion
From the original input data	17,470	4.86%	4.86% of 359,339 patients files have an identity duplication at the level of: the first and last names, birthdate and gender
After pre-processing	19,241	5.35%	1771 supplementary duplicates were detected following phase A (0.49%)
Based on the IdMR	19,241	5.35%	No collisions were detected by phases B and C

## Discussion

We have proposed a methodology to build a RD patient identifier, the IdMR, enabling patient identity federation and preventing the risk of patient re-identification. The IdMR was generated using a set of nominative data allowing stable patient identification over time and space. The data are processed to reduce variability introduced by typing errors or variations in spelling. It is a first step towards identity federation. A non-reversible hash function prevents direct re-identification. Our prerequisites were simplicity and ease of implementation. We fully tested and evaluated the approach in the context of the BNDMR project, but it could possibly be applied at the European level.

Other initiatives have been proposed, however, some of the data for the Global Unique Identifiers (GUIDs) that have been defined for RD registries may not be suitable for the generation of a unique and global identifier for all RD patients.

The NIH/NCATS Global Rare Diseases Patient Registry Data Repository, GRDR program, defined a patient identifier with the goal of being able to follow a patient across different RD studies and registries (18) (). The GRDR-GUID (global unique identifier), is a unique random alphanumeric set of characters assigned to each patient’s data. It is generated by a one-way hash algorithm upon a set of personally identifiable information: legal given name, additional name and family name at birth; day, month, and year of birth; name of city and country of birth; and finally the physical sex at birth (M/F). To our knowledge, neither the method nor the results on the robustness of this global unique identifier have yet been published. A survey [14] performed within the framework of the European joint action for RDs (RD-ACTION) [15] showed that among 19 participating countries: 10 had a unique patient identifier used mostly for drug-related groups and often not available for RD patient identification, eight did not have a unique identification system, and one built a specific identifier for rare diseases.

The Nordic countries have a long tradition of population-based health data registries, linkable on unique personal identifiers. They enable longitudinal epidemiological research with follow-up spanning many decades. Since the personal identifier is directly nominative, most often research data are distributed pseudonymized. In this case, researchers may request that a key file linking the patient identification number and the serial number be stored at a government agency responsible for the data matching, with country to country variations [16]. A recent review described the possibilities and pitfalls when combining Nordic registry data. The workload and time required to complete such cohorts should not be underestimated, the main challenges include obtaining all permissions within each country, usually in the local language, and retrieving the data [17].

Of note, Denmark, Finland, and Sweden, member of the European Union (EU), are implied in the recent EU general data protection regulation by the European Commission that enforces a unified data protection framework for all EU member states [EU General Data Protection Regulation (GDPR), https://eur-lex.europa.eu/eli/reg/2016/679/oj]. The GDPR became recently enforceable as of May 25th 2018.

The European RD-CONNECT project [18], funded by the European Union Seventh Framework Program under the International Rare Diseases Research Consortium (IRDiRC), and intended to implement a GUID, of which the GRDR-GUID is one of the possibilities under consideration.

The EpiRare project [19] defined an EU-GUID among the set of common data elements for the European Rare Disease Registration (RDR) platform. The EU-GUID is a code derived from the following data elements: patient given name, family name, sex, date and city of birth, and a unique national identification code. Neither the method nor the results for the EU-GUID have yet been published.

In our IdMR we did not include the information “city of birth” for several reasons. First, including the city of birth implied that we were able to harmonize the geographic terminologies or codes used in the source information systems. In reality, a unique standardized geographic terminology for cities does not exist either at the national (INSEE codes vs postal codes in France) or international scale (cities of all countries). Second, even if such a standard did exist, geographic terminology evolves, i.e. merging of cities (that has occurred recently in France), and could lead to different birth city entries depending on the version of the terminology in use. Third, there may be issues of granularity when using the same standard, i.e. some users could enter 75,000 for Paris and others may more precisely enter 75,001 or 75,002 to focus on a district. Finally, this information is not always available: 16% of patient files in the CEMARA database did not include the city of birth. Moreover, a quantitative study of our database showed that the four proposed nominative data types were sufficiently discriminating to avoid generating collisions. Indeed, the use of the city of birth is not necessary for avoiding collisions, and may instead be a source of errors increasing the risk of creating duplicates.

Multimodal systems for data linkage has been proposed. In the US, the SFARI initiative (Simons Foundation Austism Research Initiative) and the NDAR project (National Database for Autism Research) collaborated in building a patient identification method allowing data linkage between different autism registries [20]. A centralized system exposing web services generates GUIDs. Five identifiers can be generated after the hash of different combinations of personal data related to patients and members of their families. Using these secured web services, each source register retrieves a set of calculated identifiers according to the availability of the sent personal information and subsequently returns the anonymised patients with those GUIDs. In the central registry, data from different sources are linked, based on reconciliation between the different GUIDs.

A similar approach has been proposed for the setup of a clinical trial project allowing data linkage of six health institutions in Chicago [21]. The authors proposed a two-level system. Each institution involved in the study, at a local level, uses an application developed and distributed by the authors to generate a set of hashes of different combinations of identifying information: name, surname of birth, date of birth, social security number and gender. At a centralized level, correspondences between patients of the different institutions are inferred, based on a system of assigned coefficients to the sent hashes. This system allowed the detection of 2 million duplicates thereby reducing the total number of patient records from 7 million to 5 million. The authors stated that the system is highly efficient with a specificity of 100% and a sensitivity of 96%.

In the context of our project, the main drawback of the multimodal approaches is their complexity. On the one hand, the institutions are asked to calculate several hashes generated from identifying data that are not necessarily available in their systems, send them to the central system, retrieve the assigned identifier after seeking correspondence, integrate this identifier to records and return it all to the platform where the tests will be conducted. On the other hand, the central system must be efficient both at the technical level, to quickly operate all treatments, and at the functional level where the matching algorithm must be adapted to the source data. Moreover, the selected data for the calculation of the hashes were not relevant in the context of the BNDMR. Using the French social security number is impossible in the short-term if we are to identify children, and in the long-term if we are to identify foetuses. This latter information is potentially important in the domain of rare diseases. No existing patient identifier meets these constraints.

Patient identity management for secondary use of biomedical research data in a distributed computing environment has been proposed [22]. The ENCCA Unified Patient Identifier (EUPID) proposes a refined framework developed in the context of the European Network for Cancer research in Children and Adolescents (ENCCA). For a given patient several context-specific pseudonyms may be assigned to a virtual and inaccessible patient identifier, namely the EUPID. The identity management enables data aggregation using a hidden reference table. Phonetic hashing is proposed to prevent duplicated patient registration. Re-identification of patients is possible via a trusted third party. The architecture allows an implementation in a distributed computing environment, including cloud-based elements. Of note, an application programming interface to query the EUPID metadata is not yet available.

We deliberately chose to use specific nominative input data. A study on nominative data availability [20] showed that the most available patient information is, in the following order, the month of birth, the first name, the year of birth, the day of birth, and the last name, representing 99.6% of the patients registered during the study.

Little centralized management is necessary to sustain the reliability of the IdMR. BaMaRa is designed in a way that manages the generation of the IdMR and handles its scalability. Thus, the IdMR is generated for all new patient files, whether entered by users or sent by source systems. The four nominative data values required to generate the IdMR are also required to create a patient file in the application. A new IdMR is generated and tagged as the active one each time any of these values changes. The old IdMRs are saved in a list that tracks all the IdMR changes allowing identity federation with source systems that did not update the patient’s data. For example, patient files are sent monthly from a source system of a genetic department. Among the transferred data, a foetus file is sent. Meanwhile in BaMaRa, the same foetus was declared born with his/her own new nominative data. Using the history of IdMR changes, the received file and the existing newborn file can be federated.

The IdMR can also be implemented by source systems. It is very convenient when nominative data cannot be transferred for legal reasons. The IdMR removes, in this case, the risk of direct re-identification of patients while ensuring future identity federation.

The main constraints for the RD identifier were: sustainability, uniqueness, and non-meaningfulness. The risk of generating duplicates, one patient receiving two identifiers, is not completely avoidable due to changes that may affect the input data: first name, family name, date of birth, or gender. We identified two major causes. The first were changes affecting the nominative data, e.g. changes in the first name. This is very rare, but we recommend that source system managers notify the BNDMR team of these changes and to send the old and new IdMRs to avoid creating a new patient file in the national database. Second, errors may occur during data entry. We recommend integrating consistency and quality control measures during the data entry process.

To be implemented in different European countries, if some characters do not have a corresponding uppercase in our referential, a complementary referential is necessary. For characters from other alphabets (e.g., ñ, Å, Ø, letters which contain diacritical marks, Cyrillic or Greek characters,..) a table of correspondence with our referential will have to be implemented. In effect, if these characters were eliminated it would increase the risk of collisions. A table of equivalence using uppercases will list the characters to be transformed (i.e. for which no correspondence in our source table is available).

The risk of collisions, resulting in the same identifier for two different patients, is not inexistent. This is due to the intrinsic nature of the hash functions as there are fewer distinct potential hash codes as outputs (fixed size 256 bits) than possible input values (strings with an indeterminate length). This risk is however insignificant as the estimated probability of having a collision due to the hash algorithm sha256 is 6.9e^− 65^ for a population of 4 million people and 8.5e^− 12^ for a population^2^ of 1.4e^33^. The 20 characters threshold was set after a preliminary test on personal data of 45,000 identities. There were no collisions using an IdMR truncated to 20 characters, whereas there were six collisions when a 10 character IdMR was used. This truncation threshold may be more extensively studied for scalability purposes. Moreover, the whole string generated by the model might be stored, thus reducing the risk of collision which is already limited.

When patient data are sent by hospital information systems, the accuracy of the identifying information is high. In hospitals, patients are mostly registered using their social security card that contains inter alia the identifying information used to generate the IdMR. The identifying information for children is also stored in their parents’ card. This also works well for foetuses as it is the mother who is registered at the hospital and it is the mother’s identification information that is the most simply available to create the foetus’s IdMR. Other source systems that do not use the social security card to register patients or retrieve his/her identifying information present a potential problem. In this case, the issue of accuracy must be addressed in the data quality procedures at the national database level.

The risk of re-identification is an important security issue [23]. Hackers with access to the de-identified patient files could try to trace patient identities. The reconstruction of a mapping table linking all possible values of the nominative input data to their calculated IdMRs could be a way to re-identify patients. However, the necessary resources to build this table would be considerable, with a computation time on the order of several decades. Another approach would be to use the remaining existing information in the patient file to infer the patient’s nominative identification, even though the IdMR is an anonymised, non-significant string of characters. Indeed, it may be possible to re-identify a patient from his age, place of birth, and diagnosis, especially since the number of patients per rare disease is by definition low. To replace some of the nominative information such as first and last names by an anonymised identifier is not sufficient. The remaining information in the patient record is sensitive and must be protected. This issue is primarily addressed by the security of the information system that must imperatively be reinforced. Access rights to manage the database is an important security issue as well as exploitation rules such as those governing data aggregation or database linkage.

## Conclusion

The IdMR algorithm has two major assets: simplicity and foetus identification. The IdMR generation method is simple, easily and locally implementable by source systems. To our knowledge, foetus identification has never been addressed previously. In hospital information systems, electronic health records are usually created for the mother, even if it is the foetus that carries the disease. Moreover, the IdMR is not only a patient identifier, but may also be used for identity federation and file linkage for specific research projects, in particular RD cohorts. The simplicity of the algorithm and the universal and stable characteristics of the required input data make it potentially applicable beyond its current scope of implementation including European cross-border RD projects in the light of the recent EU Global Regulation for Data Protection.

## Footnotes

1 A collision occurs when the same IdMR is generated for two distinct patients

2 Estimation of total possible identities that could constitute inputs of the IdMR algorithm:

- All combinations of 10 characters from the Latin alphabet to form the names and surnames.
- All possible dates of birth over 100 years
- All possible genders
